# Investigating the effects of solvent polarity and temperature on the molecular, photophysical, and thermodynamic properties of sinapic acid using DFT and TDDFT

**DOI:** 10.1039/d4ra04829f

**Published:** 2024-07-24

**Authors:** Umer Sherefedin, Abebe Belay, Kusse Gudishe, Alemu Kebede, Alemayehu Getahun Kumela, Tadesse Lemma Wakjira, Semahegn Asemare, T Gurumurthi, Dereje Gelanu

**Affiliations:** a Department of Applied Physics, School of Applied Natural Sciences, Adama Science and Technology University Adama P.O. Box 1888 Ethiopia umerphysics2005@gmail.com abebe.belay@astu.edu.et; b Department of Applied Physics, School of Applied Natural and Computational Sciences, Jinka University Jinka Ethiopia; c Department of Applied Physics, College of Natural and Computational Sciences, Mekdela Amba University Tullu Awulia Ethiopia

## Abstract

Sinapic acid (SA) is widely used in cosmetics, foods, and pharmaceuticals due to its antioxidant, anti-inflammatory, neuroprotective, antimicrobial, antifungal, anticancer, and cardioprotective properties. However, environmental factors such as solvent polarity and temperature can influence its biological activity. This work determined how solvent polarity and temperature affected the molecular, photophysical, and thermodynamic properties of SA in gas and various solvents using semi-empirical (MP6), Hartree-Fock (HF) with the B3LYP method and a 6-311++G(d,p) basis set, and density functional theory (DFT) with various basis sets, such as 3TO-3G*, 3-21G+, 6-31G++G(d,p), 6-311++G(d,p), aug-CC-PVDZ, LanL2DZ, SDD, and DGD2VP. The results indicated that solvent polarity influences molecular and spectroscopic properties, such as bond angles, dihedral angles, bond lengths, FTIR spectra, solvation energy, dipole moments, HOMO–LUMO band gaps, chemical reactivity, and thermodynamic properties, resulting from interactions between the drug and solvent molecules. The findings suggested that increasing the temperature within the range of 100 to 1000 Kelvin leads to an increase in heat capacity, enthalpy, and entropy due to molecular vibrations, ultimately causing degradation and instability in SA. Furthermore, the results showed that SA underwent a redshift in the absorption peak (from 320.18 to 356.26 nm) and a shift in the fluorescence peak (from 381 to 429 nm) in the solvent phase compared to those in the gas phase. Overall, this study provides background knowledge on how solvent polarity and temperature affect the properties of SA molecules.

## Abstract 2

Sinapic acid (SA) is widely used in cosmetics, foods, and pharmaceuticals due to its antioxidant, anti-inflammatory, neuroprotective, antimicrobial, antifungal, anticancer, and cardioprotective properties.

## Graphical abstract

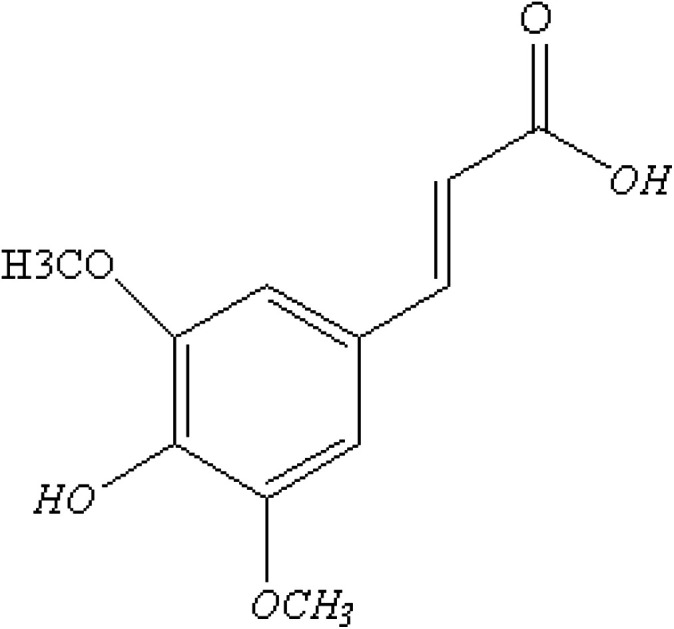

## 1 Introduction

**Fig. 1:**
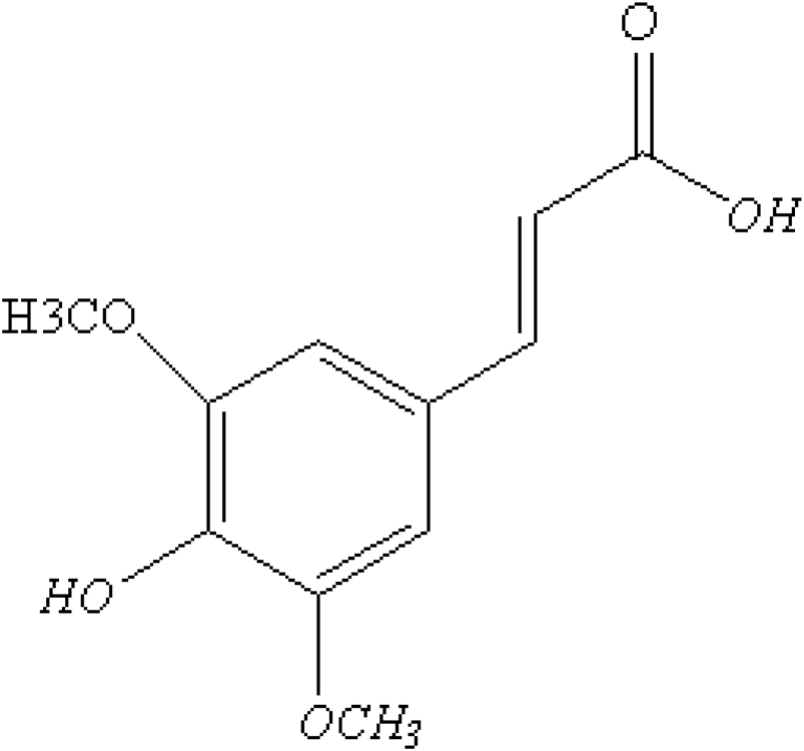
Molecular configuration of sinapic acid.

Sinapic acid (SA) is a type of hydroxycinnamic acid that contains carboxylic acid, hydroxyl, and allyl functional groups, as depicted in Fig. 1.^1^ It has a wide range of applications in food preservation,^2^ cosmetics,^3^ and pharmaceuticals.^4^ SA possesses biological activities such as antioxidant,^5^ anti-inflammatory,^6^ neuroprotective,^7^ antimicrobial,^8^ antifungal,^9^ anticancer,^10^ and cardioprotective properties.^11^ SA functions as an antioxidant by neutralizing harmful free radicals, thereby preventing cell damage and various health issues. It operates by stabilizing free radicals through the donation of hydrogen atoms or electrons. Recent research by Rostami *et al.* (2022) showcases the protective role of sinapic acid against paracetamol-induced acute liver injury by addressing oxidative stress and inflammation.^12^ This protective mechanism helps lower the risk of conditions such as inflammation,^13^ neuro-protective diseases,^14^ and cancer.^10^ In addition, it inhibits the production of reactive oxygen species (ROS) and regulates the function of antioxidant enzymes in the body to enhance antioxidant properties.^15^ Other studies have shown that the efficacy of drugs as antioxidants can be affected by temperature fluctuations^16^ and solvent polarity.^17^ Despite the antioxidant properties of SA, there were still no reports on the solvent polarity and temperature fluctuations dependent molecular, photophysical, and thermodynamic properties of the SA molecule.

Studying the effect of solvent polarity is crucial because it can influence the solubility, reaction mechanisms, and interactions between drug molecules and their targets. This factor plays a significant role in determining the thermodynamic and photophysical properties of the compounds involved. Recent study^18^ has shown that changes in solvent polarity can lead to alterations in the absorption and emission spectra, quantum efficiency, and conformational dynamics of drugs. These changes in photophysical properties can cause spectral shifts in drug molecules, indicating modifications in their electronic transitions and interactions with their surroundings, which ultimately impacts their pharmacological activity and efficacy in biological systems.^19^ Previous research has reported that the solvent can influence the photophysical properties of various pharmaceutical compounds, such as pyrazoline,^20^ isoxazole,^21^ naphthoxazole,^22^ chlorogenic acid, and caffeic acid.^21^ These results indicate that interactions between solute and solvent can affect drug photophysical properties. Furthermore, changes in solvent polarity can result in variations in the enthalpy and entropy of the drug-solvent system, thereby affecting the overall stability of the drug. Previous reports have shown changes in the thermodynamic parameters of drugs such as clozapine^23^ and ferulic acid^24^ due to solute–solvent interactions.

Temperature variation, on the other hand, has a significant effect on the structural, photophysical, and thermodynamic properties of phenolic acids, which are crucial for maintaining the stability, effectiveness, and optimal utilization of pharmaceutical products.^25^ Temperature variations can influence the stability, reactivity, and energy demands of drugs, ultimately shaping their functionality within biological systems. For example, changes in temperature can affect parameters such as heat capacity, reaction rates, and phase transitions of drugs, consequently modifying their pharmacological behavior. Previous reports have reported the temperature variation effect on the thermodynamic properties of various drugs, including polybutadiene-coated zirconia,^26^*N*-acetyl-*para*-aminophenol,^27^ ferulic acid,^24^ ipriflavone,^28^ and tinidazole.^29^ The results of the study indicate that increasing temperatures increase the molecular vibration intensities of these drugs, affecting their thermodynamic properties. Temperature fluctuations can also affect the photophysical properties, including absorption spectra and quantum efficiency of molecules. These effects primarily arise due to changes in the electronic and vibrational states of the system.^30^

Although several studies have investigated the biological effects of sinapic acid (SA) using experimental and computational methods, no reports have specifically investigated the effects of solvent polarity and temperature on its structure, thermodynamic properties, or photophysical properties. This work filled this gap by using density functional theory (DFT) to investigate the effects of solvent polarity and temperature on the structural, photophysical, and thermodynamic properties of SA molecules. The molecular structure, as shown in Fig. 1, was optimized using a semiempirical approach (MP6), Hartree–Fock (HF) with the B3LYP model and a basis set of 6-311++G (d, p) levels, and DFT with various basis sets, such as 3TO-3G*, 3-21G+*, 6-31G++G (d, p), 6- 311++G (d, p), LanL2DZ, SDD, and DGDZVP. This comprehensive approach enabled an in-depth analysis of optimized parameters (bond angles, dihedral angles, bond lengths), FTIR spectra, solvation energy, dipole moments, HOMO–LUMO band gaps, chemical reactivity, and thermodynamic properties. In addition, TD-DFT was employed to predict the absorption and fluorescence spectra based on the optimized ground state and excited state geometries, respectively.

## 2 Materials and methods

### 2.1 Materials

The molecular structure of SA was determined using ChemDraw Ultra.^31^ Computational calculations were performed with Gaussian 09W,^32^ and GaussView 6.0 (ref. 33) was used to visualize the molecular structures. Chemcraft was used to visualize the output. To investigate the molecular, photophysical, and thermodynamic properties of SA, solvents with different polarities, such as chloroform, benzene, dichloromethane, ethanol, acetone, methanol, dimethyl sulfoxide, acetonitrile, and water, were used. Temperatures ranging from 100 to 1000 K were used to study the thermodynamic parameters (heat capacity, enthalpy, and entropy) of the SA molecule.

### 2.2 Computational details

Geometry optimization of Fig. 1 was carried out utilizing a semiempirical approach (MP6), Hartree–Fock (HF) with the B3LYP method and a 6-311++G (d, p) basis set, and DFT(B3LYP) with various basis sets such as 3TO-3G*, 3-21G+*, 6-31G++G (d, p), 6-311++G(d, p), LanL2DZ, SDD, and DGDZVP. The calculations were performed with Gaussian 09 software^32^ in both gas and solvents (such as chloroform, benzene, dichloromethane, ethanol, acetone, methanol, dimethyl sulfoxide, and acetonitrile). Solvation effects were investigated using an integral equation formalism polarizable continuum model (IEFPCM).^34^ The validation process of the optimized geometries included vibrational analysis to confirm that there were no negative vibrational frequencies and to assess convergence criteria such as the RMS force, maximum force, maximum displacement, energy changes, and RMS displacement. The optimized structures were then used for various analyses, including vibrational assignment, analysis of infrared spectra, determination of thermodynamic parameters, and investigation of photophysical properties such as the HOMO, LUMO, dipole moment, and chemical reactivity of SA. The absorption spectra were obtained by TDDFT calculations on the optimized ground state geometry.^35^ The emission spectra were also determined using TDDFT calculations on the optimized excited state geometry.^24^

## 3 Results and discussion

### 3.1 The molecular optimization of sinapic acid

The molecular structure of SA was completely optimized in the gas phase using DFT and various basis sets, such as 3TO-3G*, 3-21G+, 6-31G++G (d, p), 6-311++G (d, p), LanL2DZ, SDD, and DGDZVP, to evaluate how each basis set affects the optimized structure obtained from the calculations.^36^ The dipole moment, polarizability, thermal energy, heat capacity, entropy, and HOMO–LUMO gap displayed in Table 1 were calculated using these various basis sets. The choice of basis set significantly influences the calculated molecular properties of sinapic acid, as seen in Table 1. The dipole moment, which reflects the molecule's charge distribution, ranges from 3.949 D (3TO-3G) to 5.351 D (LanL2DZ), highlighting the sensitivity of this property to the chosen basis set. Similarly, changes in the polarizability, thermal energy, heat capacity, entropy, and HOMO–LUMO gap emphasize how different basis sets affect the molecule's response to external stimuli, thermodynamic stability, and electronic structure. Among the listed basis sets, the 6-31G++G (d, p) or 6-311++G (d, p) basis sets would be suitable choices for small molecules such as ferulic acid and sinapic acid. These basis sets offer a higher level of accuracy compared to smaller basis sets like 3TO-3G* or 3-21G+*, while still maintaining reasonable computational efficiency for small molecule.

**Table 1:** Calculated dipole moment (*μ*), polarizability (*α*), thermal energy (*E*), heat capacity (*C*_p_), entropy (*S*), and HOMO–LUMO gap for sinapic acid in the gas phase by the DFT (B3LYP) methods with various basis set

Calculated parameters	Basis set						
	3TO-3G*	3-21G+*	6-31G++G (d, p)	6-311++G (d, p)	DGDZVP	LanL2DZ	SDD
*μ* (D)	3.949	5.297	4.990	4.918	4.916	5.351	5.349
*α* (a. u.)	107.788	169.221	171.922	171.809	159.537	154.973	155.145
*E* (kcal mol^−1^)	153.421	146.319	146.852	146.376	147.036	146.642	146.628
*C*_p_ (cal mol^−1^ K^−1^)	57.318	57.616	58.459	58.566	58.390	58.617	58.629
*S* (cal mol^−1^ K^−1^)	132.141	125.847	129.549	130.026	129.576	129.761	129.792
HOMO–LUMO gap (eV)	4.145	3.889	4.023	4.042	4.042	3.905	3.905

**Fig. 2:**
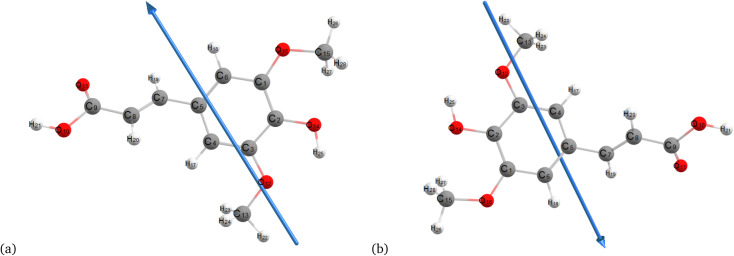
Optimization of the molecular structure of sinapic acid using semiempirical (MP6) (a) gas and (b) water.

**Fig. 3:**
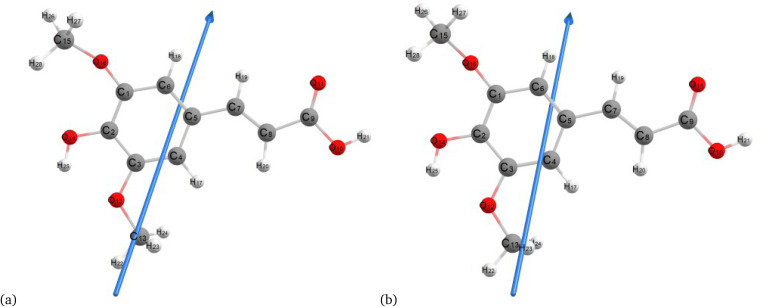
Molecular structure of sinapic acid optimized using the Hartree–Fock method with B3LYP and the 6-311++G (d, p) basis set: (a) gas and (b) water phases.

**Fig. 4:**
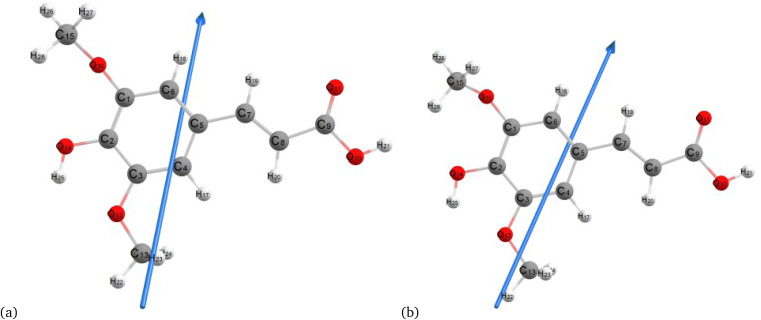
Molecular structure of sinapic acid optimized using DFT/B3LYPP with 6-311++G (d, p) (a) gas and (b) water phases.

Fig. 2–4 present the optimized structures with atom numbering obtained using semi-empirical (MP6), HF, and DFT (B3LYP) methods with the 6-311++G(d, p) basis sets, respectively, to investigate the effect of quantum calculation methods in the gas and water medium. The variations in dipole moment direction observed in gas and water phase computed by semi-empirical, Hartree–Fock, and DFT models, stem from differences in how these methods handle electronic interactions and the influence of solvent molecules on the molecular structure and dipole properties. Semi-empirical methods offer computational efficiency but sacrifice accuracy. Hartree–Fock provides a rigorous treatment of electron interactions but may overlook some electron correlation effects. On the other hand, DFT balances accuracy and efficiency, capturing electron correlation effects better than Hartree–Fock while remaining computationally manageable, making it a preferred choice for molecular structure optimization.^37^ In the gas phase, the dipole moment is inherently determined by the molecule's charge distribution.^38^ However, in water, interactions with polar solvent molecules can align the dipole moment with the surrounding solvent environment due to solvation effects.^39^

Furthermore, the bond lengths, bond angles, and dihedral angles of the molecule were calculated to analyze the optimized geometrical structure in both gas and water phases. Table 2 shows the optimized geometrical parameters (bond length, bond angle, and dihedral angle) of SA in the gas phase using HF and DFT/B3LYP methods with the 6-311++G (d, p) basis set. The HF method shows the minimum bond length between C(4)–H(17) at 1.071 Å, while the DFT method (B3LYP) indicates the maximum bond length between C(8)–C(9) at 1.476 Å. Furthermore, the HF method reveals the minimum bond angle in C(4)–C(3)–O(12) at 113.554°, while the DFT (B3LYP) method identifies the maximum bond angle in C(5)–C(4)–H(17) at 125.929°. Regarding dihedral angles, the HF method shows a minimum between C(6)–C(1)–C(2)–C(3) at 0.535° and a maximum between O(16)–C(1)–C(6)–H(18) at 179.009° according to the DFT (B3LYP) method. Dihedral angles are indicative of the spatial arrangement of atoms within a molecule, while discrepancies in bond lengths are attributed to the different levels of theory in the calculations.

**Table 2:** Calculated bond lengths, bond angles, and dihedral angles of the sinapic acid molecule in the gas phase computed by the HF and DFT (B3LYP) methods and with basis set of 6-311++G (d, p)

Bonds between atoms	Bond length (Å)		Bonds between atoms	Bond angles (°)		Bonds between atoms	Dihedral angle (°)	
	HF	DFT		HF	DFT		HF	DFT
C(1)–C(2)	1.379	1.397	C(2)–C(1)–C(6)	119.638	119.218	C(6)–C(1)–C(2)–C(3)	0.535	1.042
C(1)–C(6)	1.389	1.395	C(2)–C(1)–O(16)	121.04	122.069	C(6)–C(1)–C(2)–O(14)	−179.31	−178.106
C(1)–O(16)	1.353	1.369	C(6)–C(1)–O(16)	119.263	118.595	O(16)–C(1)–C(2)–C(3)	177.699	177.039
C(2)–C(3)	1.401	1.411	C(1)–C(2)–C(3)	119.375	119.36	O(16)–C(1)–C(2)–O(14)	−2.143	−2.109
C(2)–O(14)	1.342	1.358	C(1)–C(2)–O(14)	120.163	120.664	C(2)–C(1)–C(6)–C(5)	−0.321	−0.43
C(3)–C(4)	1.373	1.383	C(3)–C(2)–O(14)	120.463	119.97	C(2)–C(1)–C(6)–H(18)	179.009	179.126
C(3)–O(12)	1.353	1.372	C(2)–C(3)–C(4)	120.867	121.185	O(16)–C(1)–C(6)–C(5)	−177.54	−176.566
C(4)–C(5)	1.4	1.41	C(2)–C(3)–O(12)	113.554	112.881	O(16)–C(1)–C(6)–H(18)	1.794	2.989
C(4)–H(17)	1.071	1.081	C(4)–C(3)–O(12)	125.578	125.929	C(2)–C(1)–O(16)–C(15)	74.823	63.326
C(5)–C(6)	1.382	1.4	C(3)–C(4)–C(5)	119.875	119.727	C(6)–C(1)–O(16)–C(15)	−108	−120.654
C(5)–C(7)	1.47	1.457	C(3)–C(4)–H(17)	120.018	120.165	C(1)–C(2)–C(3)–C(4)	−0.407	−0.947
C(6)–H(18)	1.075	1.084	C(5)–C(4)–H(17)	120.106	120.107	C(1)–C(2)–C(3)–O(12)	−179.99	179.869
C(7)–C(8)	1.328	1.346	C(4)–C(5)–C(6)	118.964	118.777	O(14)–C(2)–C(3)–C(4)	179.434	178.208
C(7)–H(19)	1.077	1.087	C(4)–C(5)–C(7)	122.69	122.712	O(14)–C(2)–C(3)–O(12)	−0.148	−0.977
C(8)–C(9)	1.476	1.469	C(6)–C(5)–C(7)	118.346	118.511	C(1)–C(2)–O(14)–H(25)	178.336	178.909
C(8)–H(20)	1.073	1.083	C(1)–C(6)–C(5)	121.28	121.725	C(3)–C(2)–O(14)–H(25)	−1.504	−0.234
C(9)–O(10)	1.331	1.364	C(1)–C(6)–H(18)	117.892	117.757	C(2)–C(3)–C(4)–C(5)	0.053	0.208
C(9)–O(11)	1.187	1.212	C(5)–C(6)–H(18)	120.824	120.517	C(2)–C(3)–C(4)–H(17)	−179.7	−179.472
O(10)–H(21)	0.946	0.968	C(5)–C(7)–C(8)	127.816	127.902	O(12)–C(3)–C(4)–C(5)	179.582	179.28
O(12)–C(13)	1.403	1.425	C(5)–C(7)–H(19)	115.406	115.713	O(12)–C(3)–C(4)–H(17)	−0.173	−0.4
C(13)–H(22)	1.08	1.088	C(8)–C(7)–H(19)	116.779	116.385	C(2)–C(3)–O(12)–C(13)	178.194	177.797
C(13)–H(23)	1.085	1.094	C(7)–C(8)–C(9)	119.902	120.429	C(4)–C(3)–O(12)–C(13)	−1.366	−1.342
C(13)–H(24)	1.085	1.094	C(7)–C(8)–H(20)	123.763	123.163	C(3)–C(4)–C(5)–C(6)	0.167	0.414
O(14)–H(25)	0.944	0.968	C(9)–C(8)–H(20)	116.335	116.408	C(3)–C(4)–C(5)–C(7)	−179.8	−179.963
C(15)–O(16)	1.411	1.435	C(8)–C(9)–O(10)	111.665	111.254	H(17)–C(4)–C(5)–C(6)	179.922	−179.905
C(15)–H(26)	1.081	1.089	C(8)–C(9)–O(11)	126.304	126.966	H(17)–C(4)–C(5)–C(7)	−0.041	−0.282
C(15)–H(27)	1.087	1.095	O(10)–C(9)–O(11)	122.031	121.78	C(4)–C(5)–C(6)–C(1)	−0.034	−0.305
C(15)–H(28)	1.083	1.091	C(9)–O(10)–H(21)	108.468	106.643	C(4)–C(5)–C(6)–H(18)	−179.34	−179.849
			C(3)–O(12)–C(13)	120.048	118.734	C(7)–C(5)–C(6)–C(1)	179.931	−179.944
			O(12)–C(13)–H(22)	106.289	105.946	C(7)–C(5)–C(6)–H(18)	0.621	0.512
			O(12)–C(13)–H(23)	111.05	110.976	C(4)–C(5)–C(7)–C(8)	−1.12	−0.818
			O(12)–C(13)–H(24)	111.036	110.944	C(4)–C(5)–C(7)–H(19)	178.973	179.268
			H(22)–C(13)–H(23)	109.294	109.514	C(6)–C(5)–C(7)–C(8)	178.916	178.806
			H(22)–C(13)–H(24)	109.346	109.591	C(6)–C(5)–C(7)–H(19)	−0.99	−1.108
			H(23)–C(13)–H(24)	109.745	109.791	C(5)–C(7)–C(8)–C(9)	−179.94	−179.949
			C(2)–O(14)–H(25)	109.331	107.531	C(5)–C(7)–C(8)–H(20)	0.025	−0.014
			O(16)–C(15)–H(26)	106.443	105.74	H(19)–C(7)–C(8)–C(9)	−0.038	−0.035
			O(16)–C(15)–H(27)	110.5	110.298	H(19)–C(7)–C(8)–H(20)	179.93	179.9
			O(16)–C(15)–H(28)	111.116	111.426	C(7)–C(8)–C(9)–O(10)	−179.81	−179.952
			H(26)–C(15)–H(27)	109.391	109.445	C(7)–C(8)–C(9)–O(11)	0.213	0.084
			H(26)–C(15)–H(28)	109.526	109.814	H(20)–C(8)–C(9)–O(10)	0.224	0.109
			H(27)–C(15)–H(28)	109.796	110.022	H(20)–C(8)–C(9)–O(11)	−179.76	−179.855
			C(1)–O(16)–C(15)	116.583	116.86	C(8)–C(9)–O(10)–H(21)	179.976	−179.99
						O(11)–C(9)–O(10)–H(21)	−0.043	−0.024
						C(3)–O(12)–C(13)–H(22)	−179.25	−179.237
						C(3)–O(12)–C(13)–H(23)	61.975	61.972
						C(3)–O(12)–C(13)–H(24)	−60.427	−60.372
						H(26)–C(15)–O(16)–C(1)	178.929	176.951
						H(27)–C(15)–O(16)–C(1)	60.248	58.72
						H(28)–C(15)–O(16)–C(1)	−61.892	−63.78

Table 3 also shows the optimized geometric parameters of a water solution, calculated using the same quantum chemical methods and basis sets as those applied in the gas phase. In a water, hydrogen bonding interactions can influence bond lengths, angles, and dihedral angles more than can gas phase interactions. The HF method indicates that the minimum bond length between C(4)–H(17) is 1.071 Å, while the DFT (B3LYP) method also identifies this minimum distance at 1.08 Å. For maximum bond lengths, the HF method shows C(8)–C(9) at 1.474 Å, and the DFT method reports a similar maximum length for the same bond at 1.465 Å. Examining the bond angles, the HF method reveals the minimum angle between C(2)–C(3)–O(12) at 113.666°, whereas the DFT (B3LYP) method records a slightly different minimum angle of 113.028° for the same atoms. Conversely, the maximum bond angle in the HF method occurs for C(1)–C(6)–H(18) at 179.419°, and in the DFT (B3LYP) method, it is observed at 179.5° for the same atoms. Looking at the dihedral angles, the HF method shows the minimum angle in C(6)–C(1)–C(2)–C(3) at 0.336°, while the DFT method reports the minimum angle at −178.705° in C(6)–C(1)–C(2)–O(14). Similarly, the maximum dihedral angle in the HF method is observed for C(2)–C(1)–C(6)–H(18) at 179.967°, and in the DFT method, it is recorded at −179.73° for C(2)–C(1)–C(6)–H(18). The differences in bond values derived from HF and DFT methods emphasize how computational techniques influence structural optimization. HF calculations, without considering solvent effects, may show minimal changes in bond parameters, while DFT can capture solvent effects by including explicit water molecules.^40^

**Table 3:** Calculated bond lengths, bond angles, and dihedral angles of the sinapic acid molecule in water computed by the HF and DFT (B3LYP) methods with a basis set of 6-311++G (d, p)

Bonds between atoms	Bond length (Å)		Bonds between atoms	Bond angles (°)		Bonds between atoms	Dihedral angle (°)	
	HF	DFT		HF	DFT		HF	DFT
C(1)–C(2)	1.378	1.397	C(2)–C(1)–C(6)	119.887	119.582	C(6)–C(1)–C(2)–C(3)	0.336	0.648
C(1)–C(6)	1.389	1.393	C(2)–C(1)–O(16)	120.125	120.685	C(6)–C(1)–C(2)–O(14)	−179.4	−178.705
C(1)–O(16)	1.357	1.375	C(6)–C(1)–O(16)	119.947	119.641	O(16)–C(1)–C(2)–C(3)	178.029	177.164
C(2)–C(3)	1.403	1.413	C(1)–C(2)–C(3)	119.518	119.507	O(16)–C(1)–C(2)–O(14)	−1.708	−2.189
C(2)–O(14)	1.343	1.356	C(1)–C(2)–O(14)	120.014	120.295	C(2)–C(1)–C(6)–C(5)	−0.272	−0.219
C(3)–C(4)	1.374	1.384	C(3)–C(2)–O(14)	120.468	120.195	C(2)–C(1)–C(6)–H(18)	179.419	179.5
C(3)–O(12)	1.347	1.364	C(2)–C(3)–C(4)	120.557	120.771	O(16)–C(1)–C(6)–C(5)	−177.97	−176.772
C(4)–C(5)	1.402	1.411	C(2)–C(3)–O(12)	113.666	113.028	O(16)–C(1)–C(6)–H(18)	1.722	2.947
C(4)–H(17)	1.071	1.08	C(4)–C(3)–O(12)	125.777	126.198	C(2)–C(1)–O(16)–C(15)	85.414	74.051
C(5)–C(6)	1.383	1.402	C(3)–C(4)–C(5)	119.944	119.879	C(6)–C(1)–O(16)–C(15)	−96.894	−109.435
C(5)–C(7)	1.47	1.455	C(3)–C(4)–H(17)	119.799	119.853	C(1)–C(2)–C(3)–C(4)	−0.189	−0.624
C(6)–H(18)	1.075	1.084	C(5)–C(4)–H(17)	120.256	120.268	C(1)–C(2)–C(3)–O(12)	−179.98	179.95
C(7)–C(8)	1.329	1.348	C(4)–C(5)–C(6)	119.188	118.985	O(14)–C(2)–C(3)–C(4)	179.547	178.729
C(7)–H(19)	1.076	1.087	C(4)–C(5)–C(7)	122.672	122.754	O(14)–C(2)–C(3)–O(12)	−0.242	−0.697
C(8)–C(9)	1.474	1.465	C(6)–C(5)–C(7)	118.14	118.261	C(1)–C(2)–O(14)–H(25)	−179.73	179.967
C(8)–H(20)	1.073	1.082	C(1)–C(6)–C(5)	120.905	121.272	C(3)–C(2)–O(14)–H(25)	0.538	0.619
C(9)–O(10)	1.324	1.358	C(1)–C(6)–H(18)	118.314	118.264	C(2)–C(3)–C(4)–C(5)	−0.028	0.159
C(9)–O(11)	1.194	1.219	C(5)–C(6)–H(18)	120.78	120.463	C(2)–C(3)–C(4)–H(17)	−179.78	−179.538
O(10)–H(21)	0.947	0.97	C(5)–C(7)–C(8)	127.674	127.879	O(12)–C(3)–C(4)–C(5)	179.734	179.505
O(12)–C(13)	1.411	1.433	C(5)–C(7)–H(19)	115.055	115.322	O(12)–C(3)–C(4)–H(17)	−0.02	−0.193
C(13)–H(22)	1.079	1.087	C(8)–C(7)–H(19)	117.271	116.798	C(2)–C(3)–O(12)–C(13)	179.612	178.651
C(13)–H(23)	1.084	1.093	C(7)–C(8)–C(9)	120.161	120.682	C(4)–C(3)–O(12)–C(13)	−0.164	−0.738
C(13)–H(24)	1.084	1.093	C(7)–C(8)–H(20)	123.599	122.93	C(3)–C(4)–C(5)–C(6)	0.096	0.272
O(14)–H(25)	0.945	0.97	C(9)–C(8)–H(20)	116.239	116.389	C(3)–C(4)–C(5)–C(7)	−179.79	−179.92
C(15)–O(16)	1.416	1.441	C(8)–C(9)–O(10)	111.829	111.502	H(17)–C(4)–C(5)–C(6)	179.848	179.968
C(15)–H(26)	1.08	1.089	C(8)–C(9)–O(11)	126.165	126.896	H(17)–C(4)–C(5)–C(7)	−0.032	−0.224
C(15)–H(27)	1.378	1.094	O(10)–C(9)–O(11)	122.006	121.602	C(4)–C(5)–C(6)–C(1)	0.054	−0.244
C(15)–H(28)	1.389	1.092	C(9)–O(10)–H(21)	109.664	107.894	C(4)–C(5)–C(6)–H(18)	−179.63	−179.957
			C(3)–O(12)–C(13)	120.144	118.921	C(7)–C(5)–C(6)–C(1)	179.939	179.939
			O(12)–C(13)–H(22)	106.091	105.731	C(7)–C(5)–C(6)–H(18)	0.256	0.226
			O(12)–C(13)–H(23)	110.828	110.747	C(4)–C(5)–C(7)–C(8)	−1.219	−0.897
			O(12)–C(13)–H(24)	110.821	110.709	C(4)–C(5)–C(7)–H(19)	178.891	179.181
			H(22)–C(13)–H(23)	109.541	109.741	C(6)–C(5)–C(7)–C(8)	178.9	178.913
			H(22)–C(13)–H(24)	109.541	109.766	C(6)–C(5)–C(7)–H(19)	−0.99	−1.009
			H(23)–C(13)–H(24)	109.939	110.059	C(5)–C(7)–C(8)–C(9)	−179.98	179.989
			C(2)–O(14)–H(25)	109.511	107.91	C(5)–C(7)–C(8)–H(20)	−0.052	−0.087
			O(16)–C(15)–H(26)	106.707	106.123	H(19)–C(7)–C(8)–C(9)	−0.093	−0.089
			O(16)–C(15)–H(27)	110.48	110.244	H(19)–C(7)–C(8)–H(20)	179.836	179.835
			O(16)–C(15)–H(28)	110.787	111.04	C(7)–C(8)–C(9)–O(10)	179.852	179.82
			H(26)–C(15)–H(27)	109.538	109.629	C(7)–C(8)–C(9)–O(11)	−0.142	−0.168
			H(26)–C(15)–H(28)	109.584	109.719	H(20)–C(8)–C(9)–O(10)	−0.082	−0.109
			H(27)–C(15)–H(28)	109.693	110.01	H(20)–C(8)–C(9)–O(11)	179.925	179.903
			C(1)–O(16)–C(15)	115.688	115.39	C(8)–C(9)–O(10)–H(21)	−179.96	−179.933
						O(11)–C(9)–O(10)–H(21)	0.035	0.056
						C(3)–O(12)–C(13)–H(22)	−179.96	−179.76
						C(3)–O(12)–C(13)–H(23)	61.215	61.427
						C(3)–O(12)–C(13)–H(24)	−61.13	−60.938
						H(26)–C(15)–O(16)–C(1)	−179.42	−179.815
						H(27)–C(15)–O(16)–C(1)	61.583	61.544
						H(28)–C(15)–O(16)–C(1)	−60.188	−60.639

### 3.2 FTIR sinapic acid

**Fig. 5:**
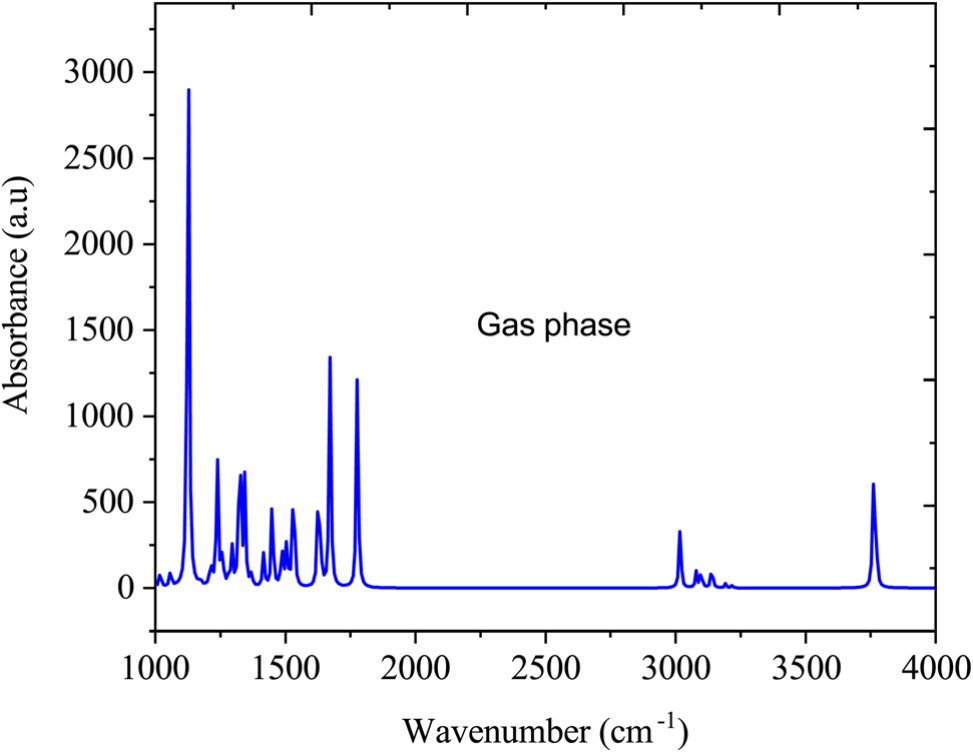
FTIR absorption spectra of sinapic acid in the gas media.

Fig. 5 shows the FTIR spectra of SA in the gas phase computed by DFT (B3LYP) with 6-311++G (d, p) basis sets. In the gas-phase FTIR spectrum, the carboxylic acid group typically exhibits a strong and broad band at approximately 1700–1725 cm^−1^, attributed to the stretching vibration of the C=O bond. The vibrations of the aromatic ring present as sharp peaks in the 1500–1600 cm^−1^ region are associated with the stretching vibrations of the C=C bonds.^41^ In addition, the O–H stretching vibration of SA is observed within the 3200–3500 cm^−1^ range, featuring a broad peak indicative of hydrogen bonding interactions.^42^

**Fig. 6:**
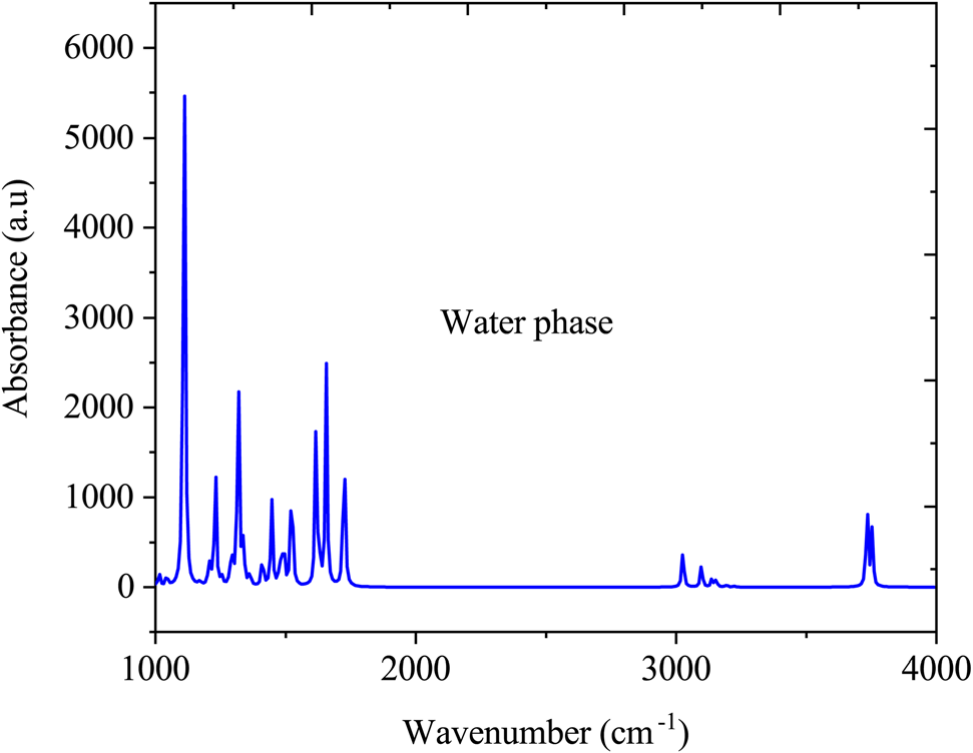
FTIR absorption spectra of sinapic acid in water media.

Fig. 6 illustrate the FTIR spectra of SA molecule in a water solvent. Similar to the gas phase, in the water phase, the O–H stretching vibration, which typically occurs between 3200 and 3500 cm^−1^, may vary in intensity and broaden due to interactions with water through hydrogen bonding. The C=O stretching vibration, typically at approximately 1700–1725 cm^−1^, may shift in the presence of water, attributed to hydrogen bonding interactions, thereby altering the vibrational frequencies. The aromatic ring vibrations of SA, which are typically found in the 1500–1600 cm^−1^ range, may undergo changes in intensity and position due to the solvation effects of water. However, the FTIR spectrum of SA dissolved in water significantly changes compared to its gas-phase spectrum due to interactions with water molecules. Water forms hydrogen bonds with the SA molecule, altering the observed vibrational frequencies and resulting in shifts in peak positions, modifications in peak intensities, and the emergence of new peaks in the FTIR spectrum, which differ from those in the gas phase.^43^

### 3.3 Energetic aspects of sinapic acid

Table 4 presents the influence of solvent polarity on the absolute energy (*E*) in Hartree and the solvation energy (*E*_solv_) in kcal mol^−1^ in different solvent polarities. Absolute energy represents the total energy state of a molecule such as electronic, vibrational, and potential energies.^44^ The values ranged from −802.7283 Hartree in the gas phase to −802.7152 Hartree in DMSO across different solvents, indicating varying levels of molecular stability. The lower the absolute energy level, the more stable the molecule, which shapes its reactivity and interactions. The solvation energy, which measures the energy change when a solute dissolves in a solvent, quantifies solute–solvent interactions.^45^ The suggested integral equation formalism polarizable continuum model (IEFPCM) was used to calculate the solvation energy in ten solvent systems, namely, benzene, dichloromethane, chloroform, ethanol, water, acetone, acetonitrile, dimethyl sulfoxide, methanol. The calculation involves a comparison of the energy values between the gas and solution phases, as outlined in Table 4. Positive (*E*_solv_) values indicate an endothermic process, while negative values suggest an exothermic reaction. The reported *E*_solv_ values range from −0.0943 kcal mol^−1^ in benzene to 0.0683 kcal mol^−1^ in DMSO, demonstrating the diverse strengths of solute–solvent interactions across different polarities. These interactions play a significant role in determining a compound's solubility and stability in specific solvents. A higher solvation energy implies stronger solute–solvent bonds, potentially influencing the compound's behavior within a solution medium.^46^ The linear correlation between solvation energy and dielectric constant indicates a strong relationship between the solvent dielectric constant (*ε*) and solvation energy*E*_solv_ = 120.3549*ε*; *R*^2^ = 0.99954

**Table 4:** Calculated bsolute energy (*E*) in Hartree, the solvation energy *E*_solv_ in kcal mol^−1^, the dipole moment in Debye, the Lippert–Mataga polarity function (*f*_LM_), and the Bakhshiev polarity function (*f*_BK_)

Media	*ε*	*n*	*E*	*E*_solv_	*μ*	*f*_LM_	*f*_BK_
Gas	—	—	−802.7283	—	4.9177	—	—
Hp	1.940	1.387	−802.7338	0.0054	5.4592	0.0021	0.0039
Bz	2.270	1.501	−802.6394	−0.0943	5.5795	0.0017	0.0036
Chl	4.810	1.446	−802.6431	0.0037	5.9684	0.1482	0.3709
DCM	8.930	1.424	−802.6451	0.0020	6.1713	0.2171	0.5903
Acetone	20.700	1.359	−802.6464	0.0014	6.3026	0.2843	0.7904
EthOH	24.500	1.361	−802.6466	0.0002	6.3226	0.2887	0.8127
MeOH	32.700	1.328	−802.6468	0.0002	6.3455	0.3086	0.8547
Acetonitrile	37.500	1.344	−802.6469	0.0001	6.3519	0.3054	0.8631
DMSO	46.700	1.479	−802.7152	0.0683	6.3675	0.3121	0.8704
Water	80.100	1.333	−802.6473	−0.0679	6.3893	0.3201	0.9136

### 3.4 Dipole moment of sinapic acid

The dipole moment (*μ*) of a molecule indicates its polarity and distribution of charges. Table 4 shows the calculated dipole moments of SA in vacuum and solvent with different dielectric constants. The electrostatic interactions between the solute and solvent become stronger, resulting in an increased dipole moment for the solute as the dielectric constant increases. The inherent polarity of SA, due to its electronegative functional groups such as hydroxyl and carboxylic acid groups, contributes to this increase. In polar solvents with high dielectric constants, such as water or dimethyl sulfoxide, these groups interact favorably with solvent molecules, aligning and stabilizing the molecule's dipole moment. This intricate relationship between the dipole moment and dielectric constant increases. The inherent polarity of SA, due to its electronegative functional groups such as hydroxyl and carboxylic acid groups, contributes to this increase. In polar solvents with high dielectric constants such as water or dimethyl sulfoxide, these groups interact favorably with solvent molecules, aligning and stabilizing the molecule's dipole moment. This intricate relationship between the dipole moment and dielectric constant reflects the dynamic interplay between molecular polarity and solvent effects. The relationship between the dipole moment and dielectric constant is*μ* = 133.3249*ε*; *R*^2^ = 0.99932

Furthermore, solvent polarity affects the Lippert–Mataga polarity function and the Bakhshiev polarity function. The *f*_LM_ polarity function focuses on the solvent-induced changes in the molecular dipole moment due to the polarity of the surrounding medium, reflecting the molecular response to solvation effects.^47,48^ On the other hand, the polarity of *f*_BK_ is dependent on solvent-induced changes in molecular polarizability, which influence the interaction of the molecule with the solvent environment.^49^ The equations corresponding to these functions are the Lippert–Mataga polarity function:^47,48^1
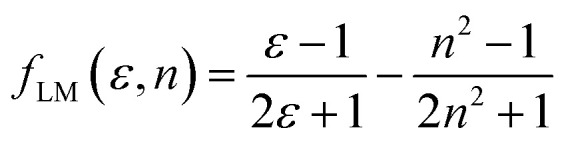


The Bakhshiev polarity function^49^ is:2
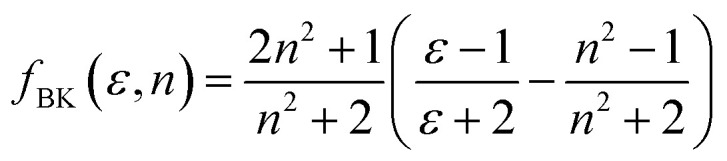


**Fig. 7:**
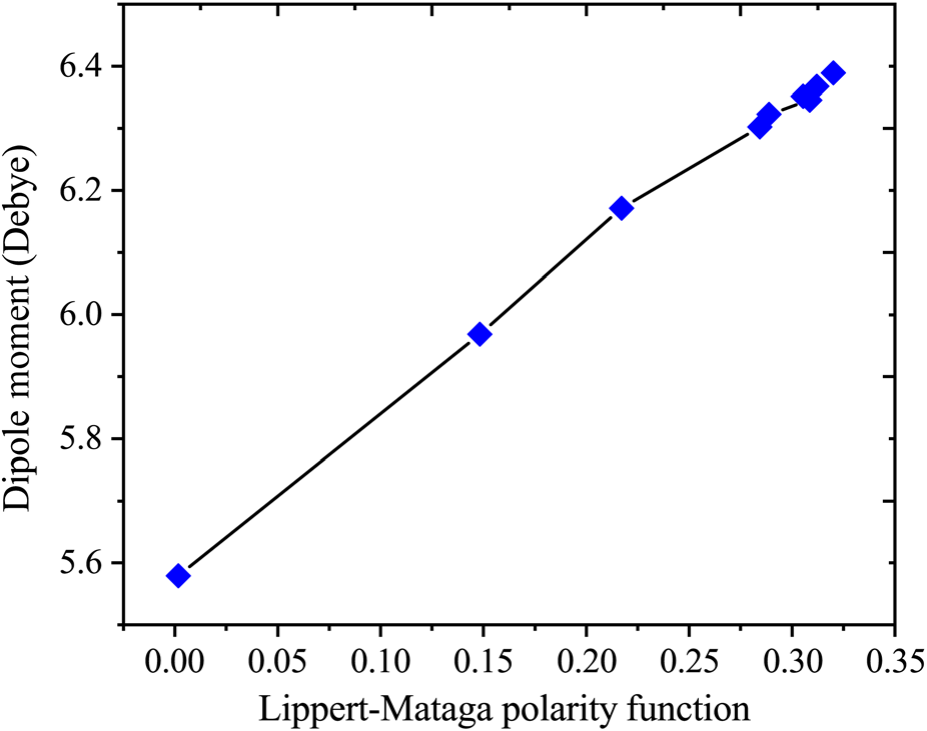
Correlations between the dipole moment and Lippert–Mataga polarity functions of sinapic acid.

**Fig. 8:**
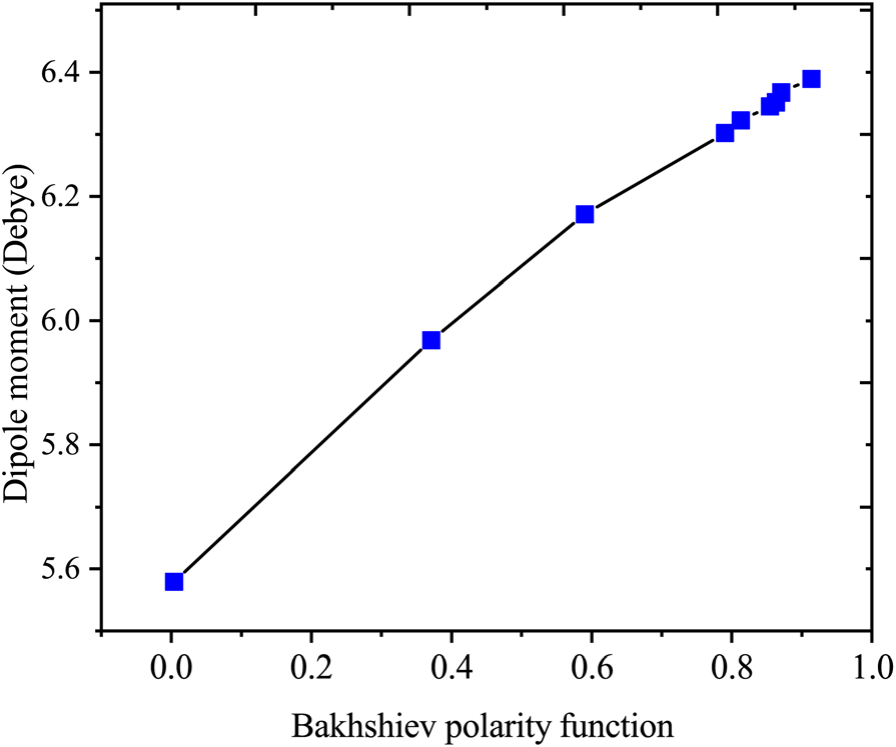
Correlations between the dipole moment and the Bakhshiev polarity function of sinapic acid.

Table 4 presents values for the Lippert–Mataga polarity function and the Bakhshiev polarity function in different solvents using eqn (1) and (2), respectively. In more polar solvents, SA displays higher *f*_LM_ and *f*_BK_ values, reflecting a stronger relationship between its dipole moment and the polarity and refractive index of the solvent. This relationship is depicted in Fig. 7 and 8 for *f*_LM_ and *f*_BK_, respectively. This suggests that sinapic acid is responsive to changes in the solvent environment, with its dipole moment adapting to the varying polarities of solvents, ultimately affecting its interactions and behavior in solution phases.

The linear correlations between *μ* and these solvent polarity functions include the following:*μ* = 2.5107*f*_LM_(*ε*, *n*) + 5.5899; *R*^2^ = 0.9936*μ* = 0.8729*f*_BK_(*ε*, *n*) + 5.5899; *R*^2^ = 0.9936

### 3.5 Thermodynamic parameters of sinapic acid

#### 3.5.1 Effect of the solvent polarity

Table 5 shows the effect of solvent polarity on the thermodynamic properties of SA. As the solvent polarity shifted from nonpolar to polar, changes in energy, entropy, and heat capacity were observed in comparison to those in the gas phase. These variations are evident in parameters such as *E*, ranging from 146.231 kcal mol^−1^ in benzene to 146.007 kcal mol^−1^ in water; *C*_p_ values fluctuating between 58.640 and 58.771 cal mol^−1^ K^−1^; and *S* values varying from 129.906 to 130.435 cal mol^−1^ K^−1^ across the solvents tested. These data underscore how the solvent environment can significantly influence the thermodynamic characteristics of SA, shedding light on its behavior in different chemical settings, which is crucial for understanding its pharmaceutical applications, ranging from drug formulation and stability to optimizing processes and improving drug efficacy.^50^

**Table 5:** Heat capacity (*C*_p_) in cal mol^−1^ K^−1^, entropy (*S*) in kcal mol^−1^, and enthalpy (*E*) in cal mol^−1^ K^−1^ for the SA calculated using DFT (B3LYP) with the 6-311++G (d, p) basis set. The solvents considered included dichloromethane (DCM), acetonitrile (MeCN), benzene (Bz), chloroform (Chl), water (W), ethanol (EtOH), dimethyl sulfoxide (DMSO), methanol (MeOH), and acetone (Ace)

Thermodynamic parameters	Gas	Non-polar			Polar					
		Bz	Chl	DCM	Ace	EtoH	MeOH	MeCN	DMSO	W
*E*	146.376	146.231	146.131	146.071	146.031	146.025	146.019	146.017	146.013	146.007
*C*_p_	58.566	58.640	58.698	58.737	58.758	58.760	58.764	58.765	58.768	58.771
*S*	130.026	129.906	130.112	130.435	130.346	130.318	130.296	130.292	130.295	130.280

#### 3.5.2 Effect of temperature

Table 6 displays the calculated enthalpy, entropy, and heat capacity values for sinapic acid at various temperatures in Kelvin for both the gas and aqueous phases, using DFT calculations with the 6-311++G (d, p) basis set. The variation in these thermodynamic properties with temperature generally increases as the temperature increases from 100 to 1000 K for sinapic acid in both the gas and water phases, indicating the effect of temperature on the energy content, heat requirements, and disorder of the substance across the temperature range studied. These findings are consistent with recent research papers discussing ferulic acid with a similar structure.^24^

**Table 6:** Enthalpy (*E*) in cal mol^−1^ K^−1^, heat capacity (*C*_p_) in cal mol^−1^ K^−1^, and entropy (*S*) in kcal mol^−1^ for the SA molecule at different temperatures (*T* in K), computed using DFT (B3LYP) with the 6-311++G (d, p) basis set

Temp (*T*)	Gas phase			Water phase		
	*E*	*C*_p_	*S*	*E*	*C*_p_	*S*
100	137.880	26.520	84.127	137.476	26.664	84.196
200	141.382	43.145	109.121	140.995	43.316	109.302
300	146.376	58.566	130.026	146.007	58.771	130.280
400	153.122	73.627	149.966	152.776	73.877	150.287
500	161.141	86.357	168.249	160.821	86.624	168.628
600	170.317	96.821	185.314	170.024	97.079	185.741
700	180.441	105.359	201.208	180.172	105.594	201.674
800	191.339	112.394	216.016	191.093	112.603	216.511
900	202.881	118.266	229.837	202.654	118.450	230.356
1000	214.962	123.224	242.771	214.752	123.384	243.307

**Fig. 9:**
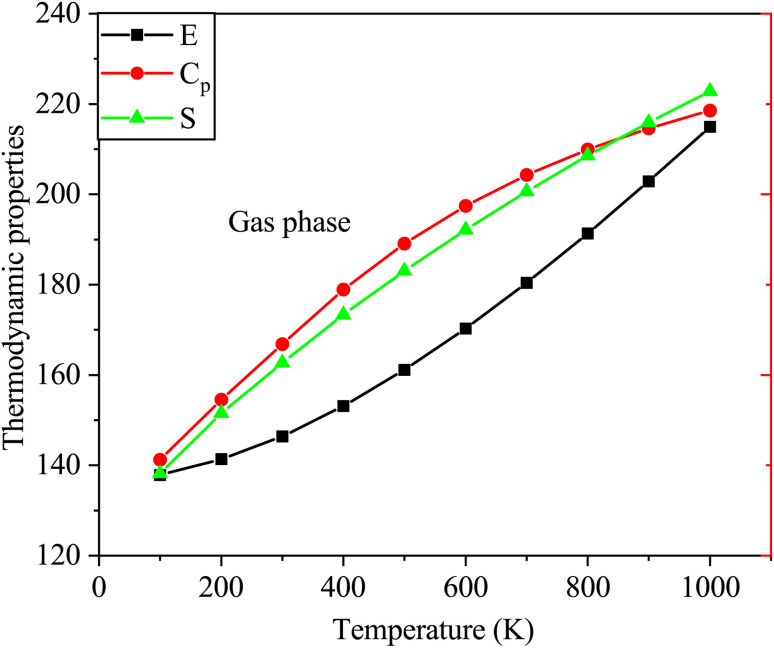
Variations in the enthalpy, entropy, and heat capacity of SA in the gas phase at temperatures from 100 to 1000 K computed by DFT employing the B3LYP functional and the 6-311++G (d, p) basis set.

Furthermore, Fig. 9 depicts the variations in thermodynamic properties such as entropy, heat capacity, and enthalpy of SA in the gas phase as a function of temperature. The calculated thermodynamic properties of SA molecule increase with increasing temperature, as shown in Fig. 9. These findings agree with results reported in prior research studies.^51^ The relationship between temperature and thermodynamic properties is modeled using a quadratic equation, yielding the following equations with high correlation coefficients (*R*^2^ ≥ 0.99944).*E* = 133.7566 + 0.0284*T* + 5.3539 × 10^−5^*T*^2^ (*R*^2^ = 0.99944)*C*_p_ = 7.1165 + 0.1987*T* − 8.3085 × 10^−5^*T*^2^ (*R*^2^ = 0.99973)*S* = 61.3359 + 0.2469*T* − 6.6103 × 10^−5^*T*^2^ (*R*^2^ = 0.99978)

**Fig. 10:**
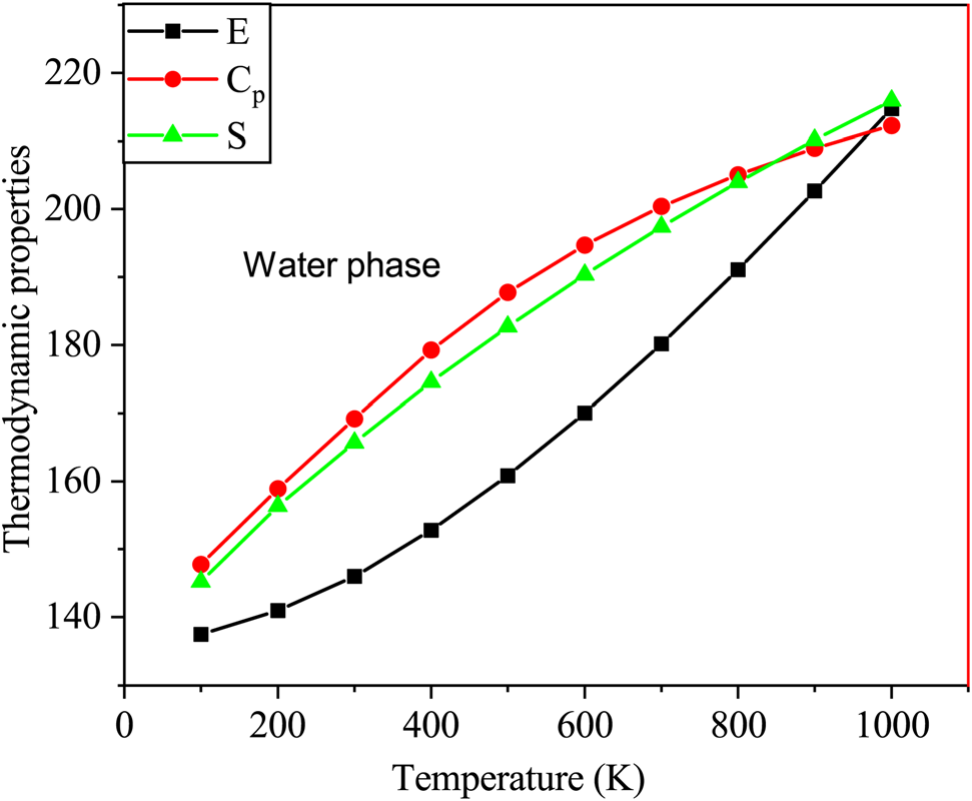
Changes in the enthalpy, entropy, and heat capacity of SA molecule in water media at a temperature range from 100 to 1000 K using DFT (B3LYP)/the 6-311++G (d, p) basis level.

Fig. 10 shows the thermodynamic properties of SA at temperatures ranging from 100 to 1000 K in the water phase. Molecular interactions in water media influence properties such as enthalpy, entropy, and heat capacity. Factors such as solvation effects, hydrogen bonding, and the polarity of the solvent in water significantly contribute to generating unique thermodynamic behaviors compared to those of the gas phase, particularly affecting entropy variations.^52^ The relationships between these thermodynamic parameters and temperature are represented by quadratic equations. This resulted in high correlation coefficients (*R*^2^ ≥ 0.99944) as follows:*E* = 133.3249 + 0.0286*T* + 5.3539 × 10^−5^*T*^2^ (*R*^2^ = 0.99944)*C*_p_ = 7.1985 + 0.1993*T* − 8.3628 × 10^−5^*T*^2^ (*R*^2^ = 0.99971)*S* = 61.3167 + 0.2479*T* − 6.6597 × 10^−5^*T*^2^ (*R*^2^ = 0.99978)

As indicated by the *R* values, gas phase alignment with heat capacity is slightly stronger than water phase alignment. On the other hand, there was no significant difference between water and gas in terms of entropy or enthalpy.

### 3.6 Molecular orbital analysis of sinapic acid

In Table 7, the calculated HOMO, LUMO, and HOMO–LUMO energies of SA in the gas phase and different solvents are presented. The highest HOMO energy is −6.138 eV in the gas phase, while the highest LUMO energy is −2.252 eV in water (W). This indicates that in water, electrons can be excited to higher energy levels, which may influence the chemical reactivity of the molecule. Generally, as the dielectric constant of the solvent increases, the HOMO and LUMO decrease. This suggests that the electron density increases in solvents with a higher dielectric constant. The largest HOMO–LUMO gap is 4.042 eV in the gas phase, indicating a greater energy difference between the highest occupied molecular orbital (HOMO) and the lowest unoccupied molecular orbital (LUMO). A larger HOMO–LUMO gap implies greater chemical stability, as more energy is required for the molecule to undergo electronic transitions, impacting its reactivity toward other molecules or reactions.

**Table 7:** Calculated HOMO, LUMO, and energy gap (in eV) using the B3LYP/6-311++G (d, p) level with the IEFPCM model in the gas phase and different solvents

Calculated parameters	Gas	Non-polar			Polar					
		Bz	Chl	DCM	Ace	EtoH	MeOH	MeCN	DMSO	W
LUMO	−2.096	−2.160	−2.204	−2.228	−2.244	−2.247	−2.249	−2.250	−2.252	−2.254
HOMO	−6.138	−6.145	−6.160	−6.170	−6.179	−6.180	−6.181	−6.182	−6.182	−6.184
LUMO–HOMO	4.042	3.985	3.956	3.943	3.935	3.933	3.932	3.932	3.931	3.930

**Fig. 11:**
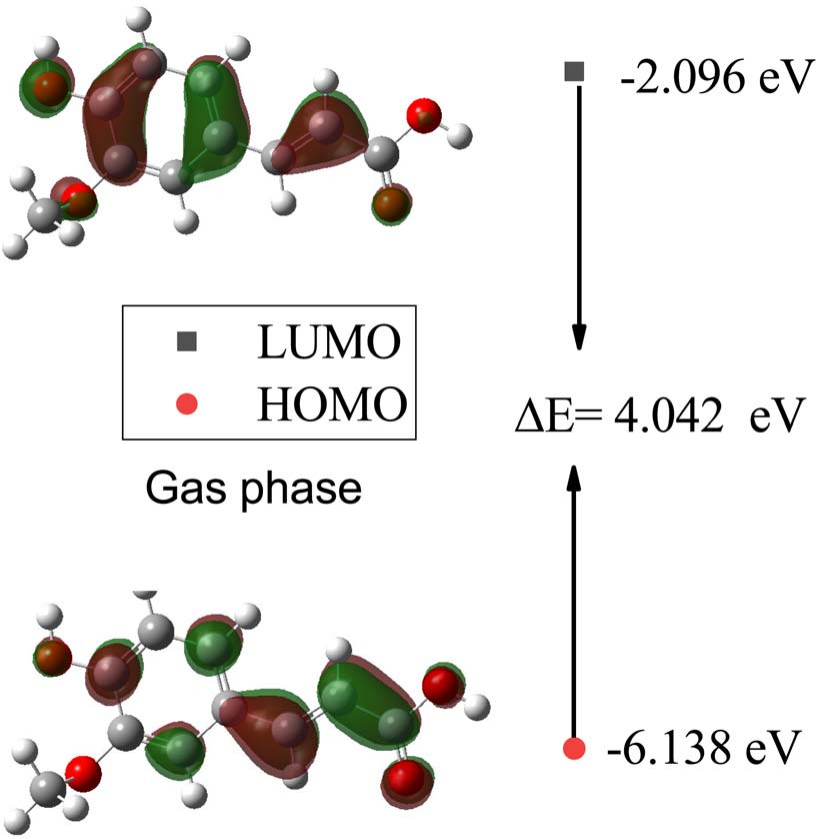
Energy levels of the HOMO, LUMO, and band gaps for SA in the gas phase computed by DFT (B3LYP) and the 6-311++G (d, p) basis set.

**Fig. 12:**
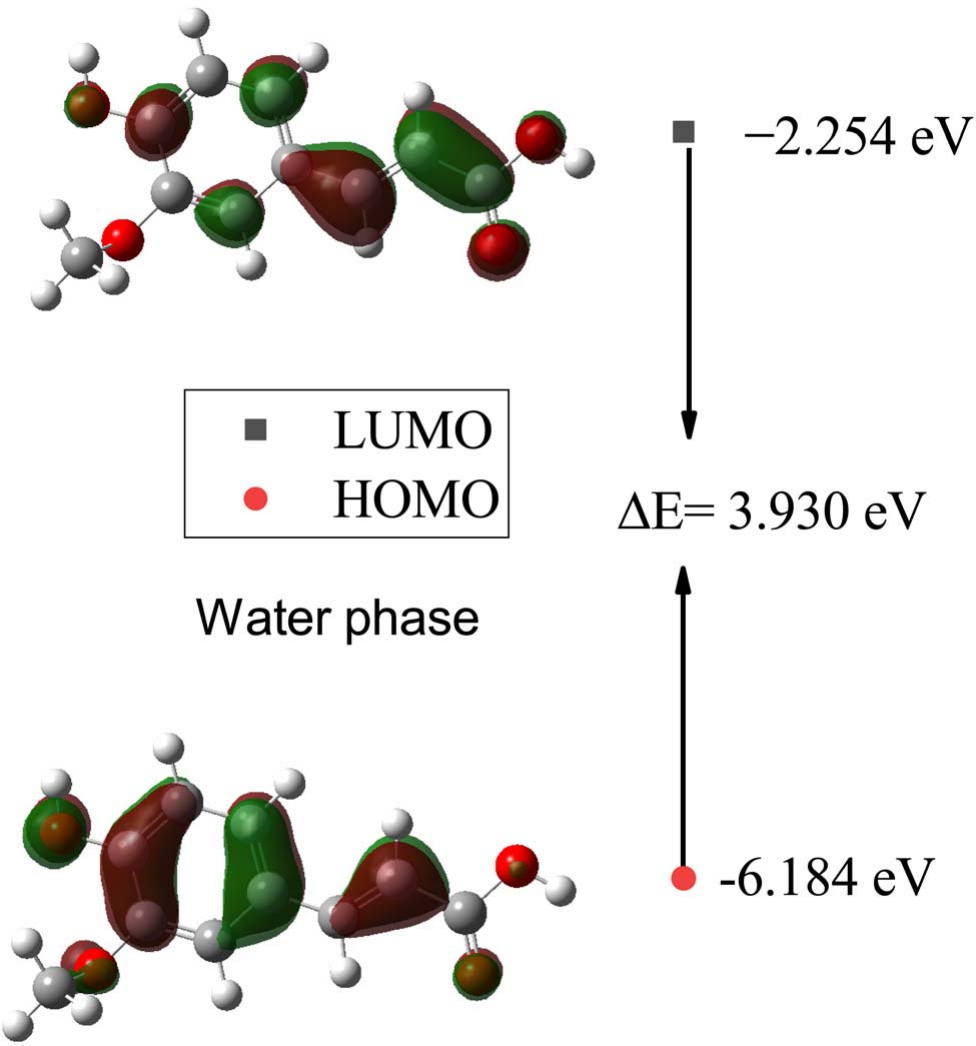
Energy levels of the HOMO, LUMO, and band gaps for SA in the water phase computed by DFT (B3LYP) and the 6-311++G (d, p) basis set.

Furthermore, plots of the HOMO and LUMO orbitals are useful for understanding the electronic structure and reactivity of molecules. The HOMO represents the highest energy level where an electron is located, while the LUMO represents the lowest energy level where an electron can be accepted.^53^Fig. 11 and 12 display the energy levels of the HOMO, LUMO, and band gaps for SA in the gas and water phases. In the gas phase, characterized by a HOMO energy of −6.138 eV and a LUMO energy of −2.096 eV, the HOMO likely represents electron-rich regions conducive to electron donation, while the LUMO indicates electron-poor sites ready for electron acceptance. The resulting 4.042 eV gap between the HOMO and LUMO signifies distinct energy levels for these orbitals in the absence of solvent interactions. In the water phase, the HOMO energy decreases to −6.184 eV, and the LUMO energy drops to −2.254 eV, indicating a shift in the electron density and reactivity within the solvated system. The reduced HOMO–LUMO gap of 3.930 eV reflects an altered electron distribution influenced by water molecules, emphasizing the impact of solvation on the electronic structure and chemical behavior of SA. The HOMO and LUMO energies differ between the gas and water phases, indicating that solvent effects can affect the chemical behavior and reactivity of molecules.^54^

### 3.7 Chemical reactivity of sinapic acid

The chemical reactivity of a drug refers to how the drug behaves in chemical reactions and interacts with other molecules, including biological targets such as enzymes, receptors, and nucleic acids.^55^ These descriptors include the ionization potential (IP),^56^ electron affinity (EA),^56^ global softness (*S*),^57^ global hardness (*η*),^58^ chemical potential (*μ*),^59^ electronegativity (*χ*),^60^ and global electrophilicity index (*ω*).^61^ These descriptors are associated with frontier molecular orbitals (FMOs). The ionization potential (IP) corresponds to the negative of the HOMO energy, indicating the energy required to remove an electron from the molecule, while the electron affinity (EA) is represented by the negative of the LUMO energy, denoting the energy released when an electron is added to the molecule. The relationship between the ionization potential (IP) and the electron affinity (EA) can be calculated by using the HOMO and LUMO energy levels, as shown in eqn (3) and (4):

3 IP = −*E*_HOMO_

4 EA = −*E*_LUMO_

Chemical reactivity parameters such as chemical hardness (*η*), chemical potential (*μ*), and electronegativity (*χ*) were calculated using (eqn (5)–(7)).^62^5
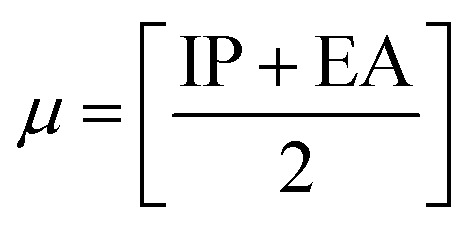
6
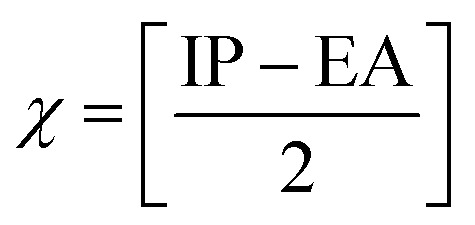
7
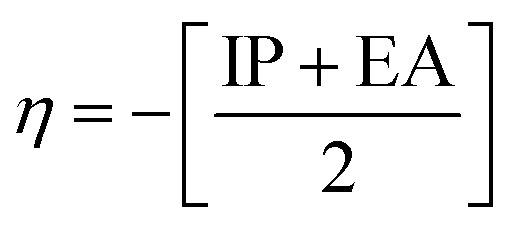


The global softness (*S*)^63^ and the global electrophilicity index (*ω*)^64^ are determined by eqn (8) and (9) respectively.8
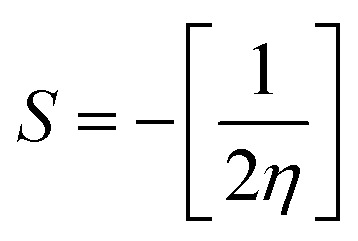
9
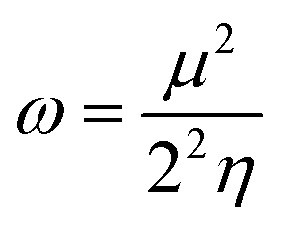


Table 8 provides a comprehensive analysis of the chemical reactivity of SA, employing various eqn (3)–(9) to calculate the reactivity. The chemical potential (*μ*) of SA increases, indicating a greater tendency for electron donation or acceptance in polar solvents. The global softness (*S*) decreases with increasing solvent polarity, suggesting reduced polarizability of the molecule in polar solvents. The electronegativity (*χ*) decreased, implying that SA has a different electron-attracting capability in polar solvents. The global hardness (*η*) shows a slight decrease, potentially affecting the stability and reactivity of SA in different solvent environments. The electrophilicity index (*ω*) increases with increasing solvent polarity, indicating the enhanced electrophilicity of SA in polar solvents. These changes highlight how solvent polarity can modulate the reactivity and behavior of SA, influencing its interactions with other molecules and its overall chemical properties.

**Table 8:** Global softness (*S*), electrophilicity index (*ω*), electronegativity (*χ*), global hardness (*η*, and chemical potential (*μ*)) of SA in gas and solvent media computed by DFT (B3LYP) with the 6-311++G (d, p) basis set

Reactivity parameters	Gas	Non-polar			Polar					
		Bz	Chl	DCM	Ace	EtoH	MeOH	MeCN	DMSO	W
EA	−2.096	−2.160	−2.204	−2.228	−2.244	−2.247	−2.249	−2.250	−2.252	−2.254
IP	−6.138	−6.145	−6.160	−6.170	−6.179	−6.180	−6.181	−6.182	−6.182	−6.184
*μ*	4.117	4.153	4.182	4.199	4.212	4.214	4.215	4.216	4.217	4.219
*η*	2.021	1.993	1.978	1.971	1.968	1.967	1.966	1.966	1.965	1.965
*S*	1.011	0.996	0.989	0.986	0.984	0.983	0.983	0.983	0.983	0.983
*χ*	−4.117	−4.153	−4.182	−4.199	−4.212	−4.214	−4.215	−4.216	−4.217	−4.219
*ω*	4.176	4.333	4.439	4.497	4.535	4.543	4.547	4.550	4.555	4.559

### 3.8 Absorption spectra of sinapic acid

**Fig. 13:**
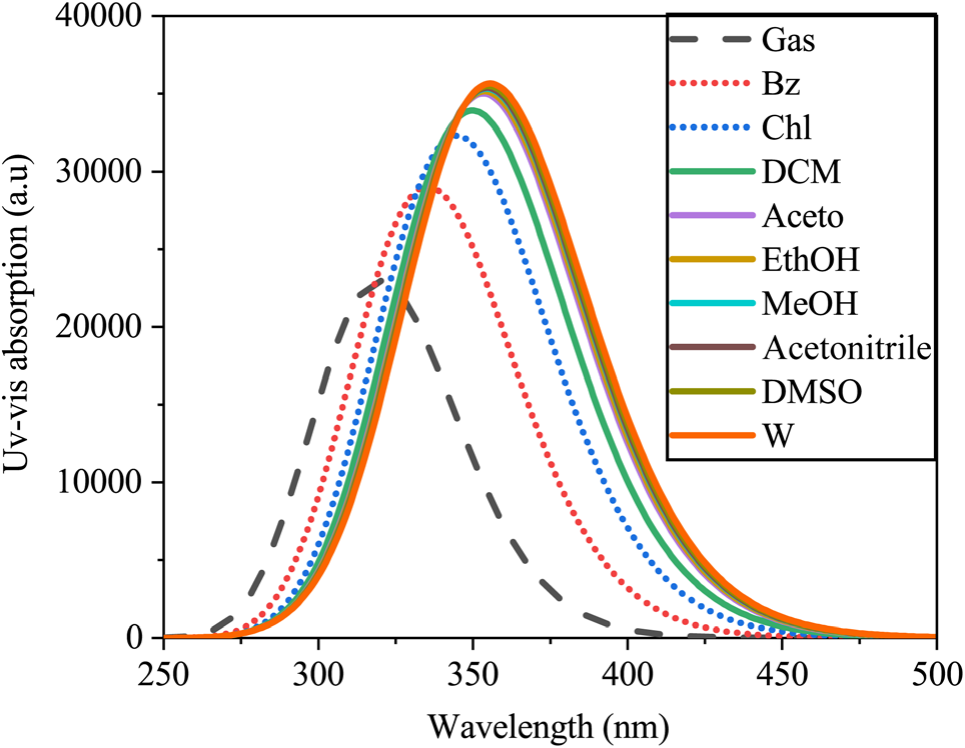
Absorption spectra of sinapic acid simulated in the gas and various solvents computed by TD-DFT/B3LYP with the 6-311++G(d, p) basis set.

The electronic absorption spectra of SA were simulated using TD-DFT with B3LYP/6-311++G (d, p), as depicted in Fig. 13.

The absorption peaks exhibited a redshift in the spectrum, ranging from 320.18 nm in water to 356.26 nm in the gas phase. This shift may be due to solvent–solute interactions, solvent polarizability disparities, and differing energy transitions within the solvent medium.^65^ The spectral data indicated the influence of solvent polarity on the electronic absorption spectra of SA, resulting in alterations in the positions, shapes, and intensities of the absorption bands. Within polar solvents such as water, interactions such as dipole–dipole or hydrogen bonding interactions may disrupt the energy levels and photon frequencies of the solute molecule, whereas nonpolar solvents such as heptane have a milder impact due to London dispersion forces.^66^ Furthermore, Table 9 presents the calculated excitation energy, maximum absorption wavelengths, and oscillator strength of SA across various solvents, illustrating how solvent effects modulate the absorption characteristics of SA. These data provide valuable insights into solute–solvent interactions, highlighting the intricate relationship between solvent properties and the electronic structure of the molecule.

**Table 9:** The wavelengths (*λ*), excitation energies (*E*), and oscillator strengths (*f*) of sinapic acid in both the gas phase and various solvents, corresponding to UV-vis spectra, were calculated by DFT (B3LYP) with a basis set of 6-311++G (d, p)

Calculated parameters	Gas	Non-polar			Polar					
		Bz	Chl	DCM	Ace	EtoH	MeOH	MeCN	DMSO	W
*E* (eV)	3.8724	3.6921	3.5897	3.5372	3.5016	3.4966	3.4909	3.4893	3.4853	3.4801
*λ* (nm)	320.18	335.81	345.39	350.52	354.08	354.59	355.17	355.33	355.73	356.26
*f*	0.5558	0.7055	0.7834	0.8226	0.8493	0.853	0.8572	0.8584	0.8613	0.8651

### 3.9 Fluorescence spectra of sinapic acid

**Fig. 14:**
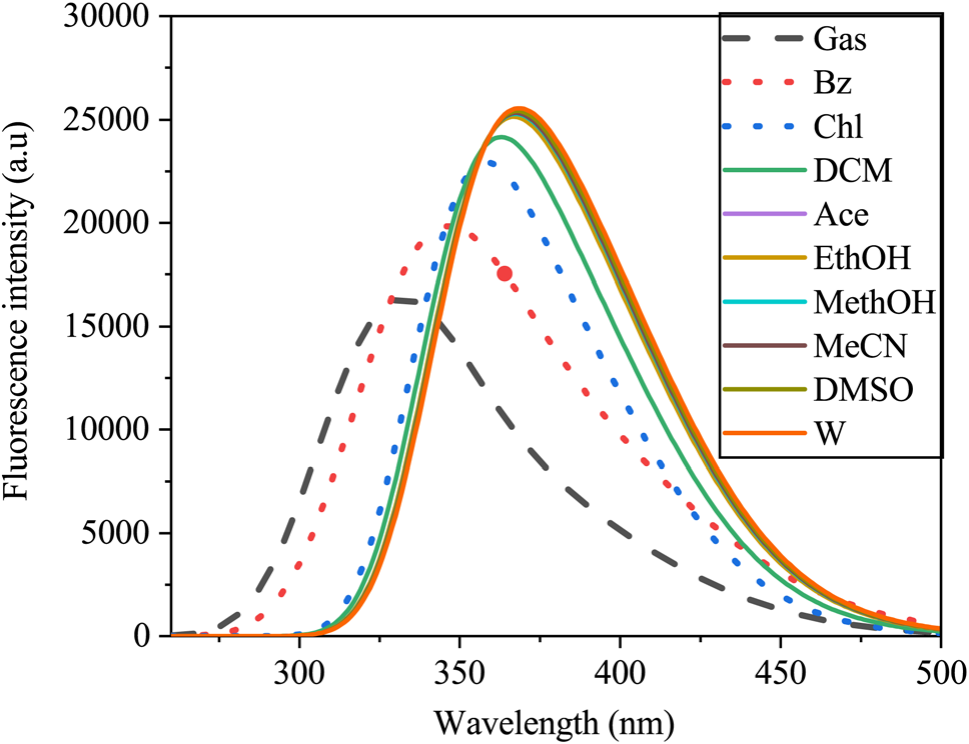
Emission spectra of sinapic acid in the gas phase and various solvents obtained *via* TD-DFT/B3LYP with the 6-311++G(d, p) basis set.

The fluorescence spectra, similar to those of the UV-vis absorption spectra, exhibited a broad single absorption band ranging from 250 to 500 nm, demonstrating sensitivity to solvent polarity (Fig. 14). As solvent polarity increased from gas to water, a redshift in the emission spectra of the SA molecule was observed. This shift, from 381 nm to 429 nm with a 31 nm increase, indicated a π–π* transition within the molecule. In gas, SA exhibits a blueshift in its spectrum compared to that in solvents. This shift may be due to the lack of solvent interactions affecting the electronic environment around the SA, leading to different emission wavelengths. In non polar solvents, the spectra may redshift compared to the gas-phase spectra due to the solvatochromic effect, which affects the polarity and electronic structure of the molecule. On the other hand, in polar solvents, the spectra could further redshift because of the enhanced solute–solvent interactions, the energy states and ultimately the emission behavior of the SA.^67^

## 4 Conclusions

This study investigated how solvent polarity and temperature influence the molecular, photophysical, and thermodynamic properties of sinapic acid. A comparison of the optimized geometric parameters for the molecule in the gas phase and in water solution revealed variations in bond lengths, bond angles, and dihedral angles between the HF and DFT (B3LYP) methods. Water solvation effects were observed in the FTIR spectrum of SA in the aqueous phase, showing shifts in peak positions, changes in peak intensities, and the appearance of new peaks due to hydrogen bonding interactions with water molecules. The dipole moment increased from gas to water, indicating stronger polarity with solvents with higher dielectric constants. In polar solvents, the HOMO–LUMO energy gap decreases compared to that in the gas phase, suggesting enhanced reactivity in these environments. UV-vis absorption and fluorescence spectra shifted toward longer wavelengths in more polar solvents, reflecting changes in electronic transitions and energy levels. The study indicated that as the solvent polarity increased, the enthalpy of sinapic acid decreased slightly, while the heat capacity and entropy increased, indicating enhanced molecular freedom and interactions in polar solvents. Temperature also influenced the thermodynamic parameters, correlating higher temperatures with increased molecular motion and disorder. This investigation provides valuable chemical insights and photophysical properties relevant for pharmaceutical development, optimizing the efficacy and stability of SA under different solvent polarities and temperatures.

## Data availability

Data for this article, including molecular configuration of sinapic acid, molecular optimization of sinapic acid, energetic and dipole moment of sinapic acid, FTIR of sinapic acid, thermodynamic parameters of sinapic acid, absorption and fluorescence spectra of sinapic acid are available at, DOI: https://doi.org/10.5281/zenodo.12771423.

## Author contributions

Umer Sherefedin: responsible for preparing inputs, conducting simulations, analyzing data, and drafting the original manuscript. Abebe Belay: involved in conceptualization, methodology development, review, editing, and supervision. Kusse Gudishe: contributed to conceptualization and provided supervision throughout the article. Alemayehu Getahun Kumela: responsible for reviewing the work and providing valuable edits. Tadesse Lemma Wakjira: engaged in simulations, reviewing the work, and making edits. Semahegn Asemare: involved in conceptualization and editing tasks. T. Gurumurthi: conducted the data analysis, reviewed the manuscript, and made necessary edits. Dereje Gelanu: engaged in simulations, reviewing the work, and making edits.

## Conflicts of interest

The authors have no conflicts of interest to declare.

## References

[cit1] Nićiforović N., Abramovič H. (2014). Compr. Rev. Food Sci. Food Saf..

[cit2] Moreno-González M., Keulen D., Gomis-Fons J., Gomez G. L., Nilsson B., Ottens M. (2021). Sep. Purif. Technol..

[cit3] Pari L., Jalaludeen A. M. (2011). Chem.–Biol. Interact..

[cit4] Raish M., Ahmad A., Ansari M. A., Alkharfy K. M., Ahad A., Khan A., Aljenobi F. I., Ali N., Al-Mohizea A. M. (2019). J. Food Drug Anal..

[cit5] Theodosis-Nobelos P., Papagiouvannis G., Rekka E. A. (2023). Antioxidants.

[cit6] Lee J.-Y. (2018). Arch. Pharmacal Res..

[cit7] Osman S. M., Soliman H. S., Hamed F. M., Marrez D. A., El-Gazar A. A., Alazzouni A. S., Nasr T., Ibrahim H. A. (2022). Pharmacophore.

[cit8] Tesaki S., Tanabe S., Ono H., Fukushi E., Kawabata J., WATANABE M. (1998). Biosci., Biotechnol., Biochem..

[cit9] Chen Z., Fang H., Hua X., Liu W., Liu Y., Xue C., Wang B., Bazhanau D., Zhu X., Yuan M. (2021). et al.. J. Chem..

[cit10] Taştemur Ş., Hacısüleyman L., Karataş Ö., Yulak F., Ataseven H. (2023). Can. J. Physiol. Pharmacol..

[cit11] Roy S. J., Prince P. S. M. (2013). Eur. J. Pharmacol..

[cit12] Rostami A., Baluchnejadmojarad T., Roghani M. (2022). Mol. Biol. Rep..

[cit13] Shahid M., Raish M., Ahmad A., Bin Jardan Y. A., Ansari M. A., Ahad A., Alkharfy K. M., Alaofi A. L., Al-Jenoobi F. I. (2022). Molecules.

[cit14] Lee H. E., Kim D. H., Park S. J., Kim J. M., Lee Y. W., Jung J. M., Lee C. H., Hong J. G., Liu X., Cai M. (2012). et al.. Pharmacol., Biochem. Behav..

[cit15] Aldubayan M. A., Ahmed A. S., Emara A. M., Ahmed A. A., Elgharabawy R. M. (2020). Oxid. Med. Cell. Longevity.

[cit16] Arina M. I., Harisun Y. (2019). Biocatal. Agric. Biotechnol..

[cit17] Mittal A., Kakkar R. (2020). Free Radical Res..

[cit18] Sherefedin U., Belay A., Gudishe K., Kebede A., Kumela A. G., Asemare S. (2024). J. Fluoresc..

[cit19] Divac V. M., Šakić D., Weitner T., Gabričević M. (2019). Spectrochim. Acta, Part A.

[cit20] Bozkurt E., Gul H. I., Mete E. (2018). J. Photochem. Photobiol., A.

[cit21] Sharma M., Pal U., Kumari M., Bagchi D., Rani S., Mukherjee D., Bera A., Pal S. K., Dasgupta T. S., Mozumdar S. (2021). J. Photochem. Photobiol., A.

[cit22] Curitol M., Ragas X., Nonell S., Pizarro N., Encinas M. V., Rojas P., Zanocco R. P., Lemp E., Günther G., Zanocco A. L. (2013). J. Photochem. Photobiol..

[cit23] Yu S., Cheng Y., Du S., Wang Y., Xue F., Xing W. (2021). J. Chem. Thermodyn..

[cit24] Sherefedin U., Belay A., Gudishe K., Kebede A., Kumela A. G., Wakjira T. L., Asemare S., Gurumurthi T. (2024). J. Mol. Liq..

[cit25] Rashid A., White E. T., Howes T., Litster J. D., Marziano I. (2014). J. Chem. Eng. Data.

[cit26] Li J., Carr P. W. (1997). Anal. Chem..

[cit27] Alauddin M. (2022). Dhaka Univ. J. Sci..

[cit28] Huang Z., Zun Y., Gong Y., Hu X., Sha J., Li Y., Li T., Ren B. (2020). J. Chem. Thermodyn..

[cit29] Li T., Zhu L., Li J., Cao Z., Sha J., Li Y., Ren B. (2022). J. Chem. Thermodyn..

[cit30] Carnero Ruiz C., Hierrezuelo J. M., Molina-Bolivar J. A. (2015). Molecules.

[cit31] Mendelsohn L. D. (2004). J. Chem. Inf. Comput. Sci..

[cit32] Frisch A. , Gaussian 09, Wallingford, USA, 2009, vol. 470, p. 25

[cit33] Medetalibeyoğlu H., Yüksek H., Özdemir G. (2019). Turk. Comput. Theor. Chem..

[cit34] Mennucci B. (2012). Wiley Interdiscip. Rev.: Comput. Mol. Sci..

[cit35] El-Shishtawy R. M., Elroby S. A., Asiri A. M., Müllen K. (2016). Int. J. Mol. Sci..

[cit36] Jalbout A., Nazari F., Turker L. (2004). J. Mol. Struct.: THEOCHEM.

[cit37] Schwöbel J. A., Ebert R.-U., Kühne R., Schüürmann G. (2011). J. Phys. Org. Chem..

[cit38] Staniforth M., Stavros V. G. (2013). Proc. R. Soc. A.

[cit39] Dill K. A., Truskett T. M., Vlachy V., Hribar-Lee B. (2005). Annu. Rev. Biophys. Biomol. Struct..

[cit40] Kamsi R. Y., Ejuh G. W., Assatse Y. T., Njeumen C. A., Tchoffo F., Ndjaka J. M. B. (2019). Chin. J. Phys..

[cit41] Sundaraganesan N., Kavitha E., Sebastian S., Cornard J., Martel M. (2009). Spectrochim. Acta, Part A.

[cit42] Singh K. S. , Majik M. S. and Tilvi S., Comprehensive Analytical Chemistry, Elsevier, 2014, vol. 65, pp. 115–148

[cit43] Howard A. A., Tschumper G. S., Hammer N. I. (2010). J. Phys. Chem. A.

[cit44] Malloum A., Fifen J. J., Conradie J. (2021). J. Mol. Liq..

[cit45] Yu H.-A., Karplus M. (1988). J. Chem. Phys..

[cit46] Mabesoone M. F., Palmans A. R., Meijer E. (2020). J. Am. Chem. Soc..

[cit47] Lippert E. (1955). Z. Naturforsch. A.

[cit48] Mataga N., Kaifu Y., Koizumi M. (1956). Bull. Chem. Soc. Jpn..

[cit49] Bakhshiev N. G., Knyazhanskii M. I., Minkin V. I., Osipov O. A., Saidov G. V. (1969). Russ. Chem. Rev..

[cit50] Parakkal S. C., Datta R., Muthu S., Alharbi N. S., Abbas G. (2023). J. Mol. Liq..

[cit51] Sultana S., Rahman M. M., Amin M. R., Rana S., Hoque M. A., Kumar D., Alfakeer M. (2021). Mol. Phys..

[cit52] Persson R. A., Pattni V., Singh A., Kast S. M., Heyden M. (2017). J. Chem. Theory Comput..

[cit53] Choudhary V., Bhatt A., Dash D., Sharma N. (2019). J. Comput. Chem..

[cit54] Elangovan N., Sowrirajan S., Arumugam N., Rajeswari B., Mathew S., Priya C. G., Venkatraman B., Mahalingam S. M. (2023). Polycyclic Aromat. Compd..

[cit55] Akintemi E. O., Govender K. K., Singh T. (2022). Comput. Theor. Chem..

[cit56] Li P., Bu Y., Ai H. (2004). J. Phys. Chem. A.

[cit57] Vela A., Gazquez J. L. (1990). J. Am. Chem. Soc..

[cit58] Roy R. K., Krishnamurti S., Geerlings P., Pal S. (1998). J. Phys. Chem. A.

[cit59] Balawender R., Geerlings P. (2005). J. Chem. Phys..

[cit60] Iczkowski R. P., Margrave J. L. (1961). J. Am. Chem. Soc..

[cit61] Pérez P. , Domingo L. R., Aizman A. and Contreras R., Theoretical and Computational Chemistry, Elsevier, 2007, vol. 19, pp. 139–201

[cit62] Yildiz D., Bozkaya U. (2016). J. Comput. Chem..

[cit63] Yang W., Parr R. G. (1985). Proc. Natl. Acad. Sci. U. S. A..

[cit64] Pearson R. G. (1986). Proc. Natl. Acad. Sci. U. S. A..

[cit65] Castro G. T., Filippa M. A., Sancho M. I., Gasull E. I., Almandoz M. C. (2020). Phys. Chem. Liq..

[cit66] Sun C. Q. (2018). Int. Rev. Phys. Chem..

[cit67] Lakowicz J. R. and Lakowicz J. R., Principles of Fluorescence Spectroscopy, 1999, pp. 185–210

